# Factors associated with knowledge, attitudes, and practices regarding food-drug interactions and drug-nutrient interactions among clinical nutrition specialists in Saudi Arabia

**DOI:** 10.3389/fpubh.2026.1914823

**Published:** 2026-09-21

**Authors:** Ohaad Fahad Awlya, Howeida Abusalih, Saad A. Alotaibi, Nadia Alghamdi, Majd Jan, Abeer Bahathig, Buthaina M. Aljehany, Nada Benajiba

**Affiliations:** 1 Clinical Nutrition Department, Faculty of Applied Medical Sciences, Umm Al-Qura University, Makkah, Saudi Arabia; 2 Department of Health Sciences, College of Health and Rehabilitation Sciences, Princess Nourah Bint Abdulrahman University, Riyadh, Saudi Arabia; 3 Department of Public Health, College of Applied Medical Sciences, Qassim University, Buraydah, Saudi Arabia; 4 Department of Public Health, College of Health Science, Saudi Electronic University, Jeddah, Saudi Arabia; 5 Department of Clinical Pharmacy and Pharmacology, Ibn Sina National College for Medical Studies, Jeddah, Saudi Arabia; 6 Department of Community Health, College of Applied Medical Sciences, Northern Border University, Arar, Saudi Arabia; 7 Food and Nutrition Department, Human Sciences and Design Faculty, King Abdulaziz University, Jeddah, Saudi Arabia; 8 Ibn Tofail University-CNESTEN, Joint Research Unit in Nutrition and Food, RDC-Nutrition AFRA/IAEA, Rabat, Kenitra, Morocco

**Keywords:** clinical nutrition specialist, drug–nutrient interactions, food–drug interactions, KAP, public health, Saudi Arabia

## Abstract

**Background:**

Food–drug interactions (FDIs) and drug–nutrient interactions (DNIs) can significantly affect therapeutic outcomes and patient safety. Clinical nutrition specialists play a key role in identifying and managing these interactions; however, their knowledge, attitudes, and practices (KAP) regarding FDIs and DNIs remain insufficiently explored in Saudi Arabia.

**Methods:**

A cross-sectional study was conducted among 353 licensed clinical nutrition specialists across Saudi Arabia. Data were collected using a validated self-administered questionnaire. Three separate multivariable linear regression models were developed to examine factors associated with knowledge, attitudes, and practices. Forest plots were generated to visualize the adjusted associations.

**Results:**

Clinical nutrition specialists had a mean knowledge score of 6.44 ± 4.08 out of 15, a mean attitude score of 31.38 ± 6.17 out of 40, and a mean practice score of 20.26 ± 5.96 out of 30. Higher knowledge scores were associated with having a doctoral degree, previous attendance at FDIs/DNIs training courses, exposure to FDIs/DNIs topics during university education, and managing patients with digestive or cardiovascular diseases. Attitude scores showed a mixed relationship with education and training: they were higher among those with a Master’s degree but lower among those with a doctoral degree or prior formal training, and were also associated with employment in the private sector, management of cardiovascular diseases, and knowledge scores. Better practice scores were associated with higher knowledge and attitude scores, whereas a longer time since graduation was associated with lower practice scores; neither educational attainment nor formal training remained independently associated with practice scores in the adjusted model.

**Conclusion:**

Saudi clinical nutrition specialists demonstrated positive attitudes but had important knowledge and practice gaps regarding FDIs and DNIs. Higher educational attainment and formal training were consistently associated with greater knowledge, but their relationship with attitudes was inconsistent, and neither was independently associated with practice. These domain-specific patterns highlight the importance of strengthening undergraduate curricula, pairing continuing professional development with attention to its effect on practitioner attitudes, and improving access to clinical decision-support tools.

## 1 Introduction

The combined use of diet and pharmacotherapy represents a cornerstone of modern healthcare, particularly in the prevention and management of chronic diseases. However, this coexistence may lead to clinically significant interactions, commonly referred to as food–drug interactions (FDIs) and drug–nutrient interactions (DNIs), which are two related but distinct phenomena: FDIs occur when dietary components influence the action, bioavailability, or safety profile of medications, whereas DNIs arise when drugs interfere with nutrient intake, absorption, or metabolism (1–3). These two concepts are sometimes used interchangeably in the literature, and this distinction is maintained consistently throughout this manuscript. As the global burden of chronic diseases increases, along with the widespread use of polypharmacy, these interactions have become increasingly relevant in clinical practice. Reported FDIs prevalence varies considerably across settings and methodologies rather than converging on a single figure. For example, a study conducted in a Brazilian public hospital found that 19.5% of prescribed medications had a potential food interaction (4), while a cross-sectional study of 400 inpatients in Northeast Iran found that 89.8% of patients were taking at least one medication at an inappropriate time relative to meals (5). This variability illustrates that FDIs prevalence depends heavily on the drug classes studied, the definition of a “potential interaction” used, and the clinical setting.

Despite the clinical and economic relevance of FDIs and DNIs, evidence on healthcare professionals’ knowledge, attitudes, and practices (KAP) regarding these interactions remains limited, and this gap is particularly pronounced among clinical nutrition specialists, who are directly responsible for nutritional counseling for patients receiving pharmacotherapy. In Saudi Arabia specifically, no study to date has systematically assessed clinical nutrition specialists’ KAP regarding FDIs and DNIs, despite the expanding scope of dietetic practice and the growing integration of nutrition services within the healthcare system.

From a mechanistic perspective, FDIs and DNIs may involve both pharmacokinetic and pharmacodynamic pathways. Pharmacokinetic interactions typically affect gastrointestinal absorption, hepatic metabolism, or renal excretion, while pharmacodynamic interactions modify the physiological response to medications without necessarily altering their concentration (6, 7). Several clinically relevant examples illustrate the significance of these interactions: foods rich in tyramine may trigger severe hypertensive reactions in patients treated with monoamine oxidase inhibitors, variability in dietary vitamin K intake can destabilize anticoagulant control with warfarin (8, 9), dairy products may reduce the absorption of tetracyclines through chelation, and grapefruit is known to inhibit intestinal cytochrome P450 enzymes, increasing systemic exposure to several medications (10).

Long-term use of commonly prescribed medications has also been associated with deficiencies in essential micronutrients. In a secondary analysis of the Diabetes Prevention Program Outcomes Study involving over 2,000 participants, long-term metformin use was associated with low or borderline-low vitamin B12 levels in 19.1% of users at 5 years (versus 9.5% among non-users) and 20.3% at 13 years (versus 15.6% among non-users) (11). Chronic proton pump inhibitor (PPI) use has similarly been linked to impaired absorption of vitamin B12, magnesium, calcium, and iron (12). A recent retrospective study found that the prevalence of deficiencies varies substantially by PPI agent, with magnesium deficiency ranging from 11.9% among omeprazole users to 39.3% among esomeprazole users, and vitamin B12 deficiency ranging from 38.6 to 59.0% across PPI types (13).

Adverse drug events related to medication interactions are estimated to account for approximately 5–10% of hospital admissions worldwide, a significant proportion of which are considered preventable through appropriate screening, counseling, and monitoring (14). Multidisciplinary interventions involving clinical nutrition specialists, pharmacists, and physicians can reduce interaction-related complications and improve medication safety (15). Within this context, clinical nutrition specialists are uniquely positioned, through nutritional assessment, patient education, and collaboration within multidisciplinary teams, to identify potential interactions and provide tailored dietary recommendations (3).

Nevertheless, knowledge and practice related to FDIs and DNIs remain suboptimal among healthcare professionals more broadly. In Saudi Arabia, fewer than half of healthcare professionals demonstrate adequate knowledge of FDIs, a gap often attributed to insufficient training and limited integration of clinical nutrition into professional education (16, 17). Among the general population, more than 60% lack sufficient knowledge about potential interactions between food and medications (18, 19). Research focusing specifically on clinical nutrition specialists, however, remains scarce, particularly regarding their knowledge, attitudes, and practices (KAP) related to FDIs and DNIs, which constitutes the central gap this study addresses.

Given the expanding scope of dietetic practice in Saudi Arabia, this study aimed to assess clinical nutrition specialists’ KAP regarding FDIs and DNIs, and to examine factors associated with KAP outcomes, including level of education, years of experience, and prior training. Beyond descriptive KAP estimates, multivariable regression analysis was conducted to determine whether these associations persisted after adjusting for potential confounding professional characteristics, given the cross-sectional design of the study. For this study, a clinical nutrition specialist was defined as a healthcare professional holding at least a bachelor’s degree in clinical nutrition, licensed by the Saudi Commission for Health Specialties, and actively engaged in direct patient care.

## 2 Methods

### 2.1 Study design and participants

This study was a national online convenience survey conducted between January and November 2024, covering all five administrative regions of Saudi Arabia. Ethical approval was obtained from the Human Research Ethics Committee of the Faculty of Medicine at King Abdulaziz University, Jeddah, and the Ministry of Health (Approval No. A01935). The participants were licensed clinical nutrition specialists who self-reported actively providing patient care in Saudi Arabia. Both male and female professionals with a valid license from the Saudi Commission for Health Specialties (SCFHS) were eligible. Clinical nutrition specialists working in hospitals, clinics, academic institutions, online nutrition services, or self-employed practitioners were included, provided they self-reported that their role involved direct clinical nutrition care. Those not involved in direct patient care or lacking a valid SCFHS license were excluded.

Participants were identified and invited through open digital dissemination: the survey link was shared via WhatsApp groups of clinical nutrition specialists, Telegram channels, professional email lists, and social media platforms (Twitter/X, LinkedIn). All participants received detailed information about the study objectives, confidentiality, and procedures. Electronic informed consent was obtained before enrollment. Participation was voluntary, and participants could withdraw at any time. No incentives were provided, and all responses were collected anonymously.

### 2.2 Sample size

The total number of registered clinical nutrition specialists in Saudi Arabia was reported as 4,144 by the Saudi Commission for Health Specialties (SCFHS) in 2018 (20). Assuming a 50% expected prevalence of good practice, a 95% confidence level (*Z* = 1.96), and a 5% margin of error, the initial required sample size (*n*₀) was calculated as 384 participants. Because the target population (*N* = 4,144) was finite, a finite population correction (FPC) was applied using the following formula: *n* = *N*₀/[1 + (*N*₀ − 1)/*N*], Substituting *N*₀ = 384 and *N* = 4,144 gives *n* = 384/[1 + 383/4,144] ≈ 352, which is the minimum sample size reported.

### 2.3 Data collection

A previously validated questionnaire (21) was adapted for this study. Minor modifications were made to ensure contextual relevance and clarity. Specifically, items deemed not applicable to the target population were removed, multiple-choice questions were converted into single-best-answer formats to reduce ambiguity, and the attitude scale was expanded from three to five points to improve sensitivity and capture nuanced perceptions. In addition, two open-ended practice questions were transformed into closed-ended items to facilitate quantitative analysis.

Translation and back-translation were not undertaken, as the questionnaire was developed and administered in English. This approach was appropriate because English is the language of instruction for clinical nutrition education in Saudi Arabia at both undergraduate and postgraduate levels. Furthermore, all participants were licensed clinical nutrition specialists who had completed their professional education in English and regularly used English scientific and clinical terminology in their routine practice. Therefore, the questionnaire language was considered appropriate for the target population, minimizing the risk of misinterpretation of technical concepts.

The final questionnaire was administered online using Google Forms and disseminated through multiple digital platforms to maximize reach and participation. The survey was distributed through an open online recruitment strategy using professional networks and social media platforms. As the total number of eligible clinical nutrition specialists who received or viewed the survey invitation was unknown, a response rate could not be calculated. The Google Forms survey was configured to accept only one response per Google account (without collecting email addresses), thereby minimizing duplicate submissions while preserving participant anonymity. Additionally, the dataset was screened during data cleaning for potential duplicate entries based on response timestamps and participant characteristics, and no duplicate responses were identified. Participation was voluntary and anonymous.

The questionnaire consisted of four sections:

1. Demographic and Professional Background: This section collected detailed information on participants’ characteristics, including age, gender, nationality, geographical region of practice, year of graduation, years of professional experience, highest academic qualification, type of workplace (government or private), and prior exposure to nutrition- or FDIs/DNIs related training. Information on clinical practice areas (e.g., endocrine, cardiovascular, digestive, and other diseases) was also collected, as these variables were considered potential factors associated with KAP.
2. Knowledge: The knowledge section comprised 15 items addressing clinically relevant FDIs and DNIs commonly encountered in dietetic practice. Topics included well-established interactions such as vitamin K–warfarin, grapefruit–cardiovascular drugs, potassium–ACE inhibitors, and drug-induced effects on nutrient absorption. Each item had one correct answer, and responses were coded as 1 (correct) and 0 (incorrect or “I do not know”) to minimize guessing bias. Total knowledge scores ranged from 0 to 15, with higher scores indicating better knowledge.
3. Attitudes: Attitudes were assessed using eight statements designed to evaluate participants’ perceptions of the clinical importance of FDIs/DNIs, their perceived professional responsibility, and the need for awareness and education. Responses were measured on a five-point Likert scale ranging from “strongly disagree” (1) to “strongly agree” (5). All eight items, including the item addressing whether FDIs differ from DNIs, were worded and scored in the same direction, with no items reverse-coded. Total scores ranged from 8 to 40, with higher scores reflecting more positive attitudes.
4. Practices: The practice section included six items assessing the frequency of behaviors related to FDIs/DNIs in routine clinical practice. These behaviors included patient medication history taking, counseling, use of information resources, referral or documentation of interactions, and engagement in continuing education. Responses were recorded on a five-point Likert scale ranging from “never” (1) to “always” (5). Total scores ranged from 6 to 30, with higher scores indicating better practices.

### 2.4 Validity and reliability

The questionnaire was adapted from a previously validated instrument developed by Sultan et al. (21) to better align with the objectives of the present study (22, 23). The modifications included revising item wording for clarity, updating the knowledge section to reflect current evidence on FDIs and DNIs, and restructuring some response options while retaining the original domains of knowledge, attitudes, and practices. Because these modifications could affect the content validity and comparability of the instrument, the adapted questionnaire underwent a new face and content validation process before data collection, consistent with recommendations for modified survey instruments (22, 24).

Face validity was assessed by a multidisciplinary panel of nine experts in clinical nutrition, pharmacology, and survey methodology. Each expert independently evaluated the questionnaire using a standardized assessment form that addressed the instrument’s comprehensiveness, clarity, relevance, and feasibility. Subsequently, the research team reviewed all quantitative ratings and qualitative comments. Suggestions for modification were discussed among the investigators, and consensus was reached by considering both the level of agreement among the experts and whether the proposed changes improved clarity or contextual relevance without changing the construct being measured. Revisions were implemented only when supported by the expert feedback and the overall content validity assessment. Overall, 80% of experts agreed that the questionnaire adequately covered the intended content, 100% considered the items understandable, and the mean rating for questionnaire completion time was 9.4 ± 1.3 on a 10-point scale.

The content-validity assessment involved nine experts, all of whom rated the 29 questionnaire items using the five-point relevance scale. Ratings of 4 or 5 were considered evidence of item relevance. Twenty-eight items received ratings of 4 or 5 from all nine experts, resulting in an I-CVI of 1.00 for these items. For the remaining item, 8 of 9 experts provided a rating of 4 or 5, resulting in an I-CVI of 0.89. The resulting S-CVI/Ave was 1.00, calculated as [28(1.00) + 0.889]/29 = 0.996, and the S-CVI/UA was 0.97 (28/29 = 0.966). exceeding the recommended thresholds for excellent content validity (24). These findings indicate that the adapted questionnaire demonstrated excellent content validity and was considered appropriate for assessing clinical nutrition specialists’ knowledge, attitudes, and practices regarding FDIs and DNIs.

Internal consistency reliability was assessed using Cronbach’s alpha based on a pilot study involving 22 licensed clinical nutrition specialists, who were not included in the final study sample. The knowledge, attitudes, and practice domains demonstrated good internal consistency, with Cronbach’s alpha coefficients of 0.81, 0.85, and 0.79, respectively. As the knowledge domain items were scored dichotomously (correct/incorrect), the reported coefficient (α = 0.81) is mathematically equivalent to the Kuder–Richardson Formula 20 (KR-20), which is a special case of Cronbach’s alpha for binary-scored items. The overall questionnaire yielded a Cronbach’s alpha of 0.75, indicating acceptable internal consistency (25). Overall instrument reliability was considered satisfactory for research purposes (26). The development and adaptation of the questionnaire followed established methodological guidelines for instrument design (27).

### 2.5 Statistical analysis

Data were analyzed using R software version 4.4.0 (R Foundation for Statistical Computing, Vienna, Austria). Descriptive statistics (frequencies and percentages) summarized participant characteristics. The normality of continuous outcome variables was assessed using visual inspection of histograms and Q-Q plots together with the Shapiro–Wilk test. Given the relatively large sample size, parametric tests were considered robust to minor deviations from normality. Comparisons of mean knowledge, attitude, and practice scores across participant characteristics were performed using Welch’s independent-samples t-test (which does not assume equal variances between groups) for comparisons between two groups, or one-way analysis of variance (ANOVA) for comparisons among three or more groups. To further investigate the factors associated with KAP scores, separate multivariable linear regression models were constructed for each outcome variable. Before model building, univariate analyses were performed to assess the association between each independent variable and the respective outcome. Variables with a *p*-value < 0.20 in univariate analysis were considered eligible for inclusion in the initial multivariable models to avoid excluding potentially important predictors. A backward stepwise selection approach was subsequently applied to refine each model. Variables were sequentially removed based on likelihood ratio tests comparing nested models. Specifically, a variable was excluded if its removal did not significantly worsen model fit (*p* > 0.20) and did not result in more than a 10% change in the estimated coefficients of the main exposure variables, thereby minimizing the risk of residual confounding. Although stepwise selection methods may be sensitive to sampling variation, their potential instability was mitigated by prespecifying candidate variables based on univariate screening (*p* < 0.20), applying likelihood ratio tests and change-in-estimate criteria during backward elimination, assessing multicollinearity, and verifying the adequacy of the final models through diagnostic analyses. Multicollinearity among independent variables was assessed using variance inflation factors (VIF), with values greater than 5 indicating potential collinearity concerns. The final models were evaluated for overall goodness-of-fit and compliance with regression assumptions. Linearity between continuous predictors and outcomes was examined, while homoscedasticity and normality of residuals were assessed through visual inspection of residual plots and quantile–quantile (Q–Q) plots. Influential observations were identified using standardized residuals and leverage statistics. Regression coefficients (*β*) with corresponding 95% confidence intervals (CI) were reported to quantify the strength and direction of associations. Full initial and final model specifications, including standard errors, 95% confidence intervals, exact *p*-values, reference categories, analytic sample sizes, adjusted *R*^2^, and variance inflation factors for every model, are reported in Supplementary Tables S1–S3. All statistical tests were two-sided, and a *p*-value < 0.05 was considered statistically significant.

## 3 Results

### 3.1 Socio-demographic characteristics of the studied population

This study included 353 clinical nutrition specialists, with a marked predominance of female participants (83.57%). The age distribution highlights a relatively young workforce, with the majority aged 23–29 years (57.22%), followed by those aged 30–39 years (35.69%). More than two-thirds (69.41%) reported 0–4 years of practice, suggesting that the sample largely consists of early-career clinical nutrition specialists. In terms of nationality, the sample was predominantly Saudi (92.07%), with higher representation from the Western (37.68%) and Central (26.91%) regions. Regarding educational attainment, the majority held a bachelor’s degree (80.45%), with fewer participants having postgraduate qualifications (Master’s: 15.58%; Doctorate: 3.97%). Although 71.10% of participants reported that their university education included topics related to FDIs and DNIs, only 41.08% had attended additional training courses. A considerable proportion either had not attended such training (44.19%) or could not recall (14.73%). Most participants graduated within the last five years (60.06%), and the majority obtained their degrees from Saudi institutions (87.82%). From a professional perspective, more than half of the participants worked in the government sector (57.22%), while 42.78% were employed in the private sector. The most managed conditions, reported as multiple-response items, so percentages do not sum to 100%, included (74.50%), endocrine diseases such as diabetes (73.37%), cardiovascular diseases (67.14%), and digestive disorders (66.01%). In contrast, less frequently reported areas included mental health (17.28%) and inherited metabolic disorders (24.08%) (see Table 1).

**Table 1:** Socio-demographic characteristics of the studied population (*N* = 353) (*n*, %).

Variables	*N*	%
Gender		
Male	58	16.43
Female	295	83.57
Age (years)		
23–29 years	202	57.22
30–39 years	126	35.69
40–49 years	23	6.52
50 years and above	2	0.57
Nationality		
Saudi	325	92.07
Non-Saudi	28	7.93
Geographical area		
Western	133	37.68
Eastern	51	14.45
Southern	35	9.92
Central	95	26.91
Northern	39	11.05
Educational qualification		
Bachelor’s	284	80.45
Master’s	55	15.58
Doctorate	14	3.97
Graduation year		
Within the last 5 years	212	60.06
5–10 years ago	86	24.36
More than 10 years ago	55	15.58
Graduation university		
Saudi Arabia	310	87.82
Non-Saudi	43	12.18
Type of workplace sector		
Government	202	57.22
Private	151	42.78
Years of Experience as a clinical nutrition specialist		
0–4	245	69.41
5–9	60	17.00
10–14	35	9.92
15–19	9	2.55
Over 20	4	1.13
Have you attended courses on food-drug interactions and Nutrient-drug interaction?		
Yes	145	41.08
No	156	44.19
I do not remember	52	14.73
Did your university studies include subjects related to food-drug interactions and nutrient-drug interaction?		
Yes	251	71.10
No	65	18.41
I do not remember	37	10.48
What diseases do you manage in your clinical practice as a clinical nutrition specialist?*		
Digestive Diseases such as Peptic Ulcer, Ulcerative Colitis, IBS, Celiac disease	233	66.01
Endocrine Diseases such as (Diabetes, thyroid)	259	73.37
Cardiovascular Diseases such as (Hypertension, …)	237	67.14
Inherited metabolic disorders	85	24.08
Food allergies	214	60.62
Kidney disease	215	60.91
Mental health	61	17.28
Obesity	263	74.50
Cancer	107	30.31

^*^Disease categories were multiple responses.

### 3.2 Knowledge, attitude and practice

Figure 1 presents the percentage of correct responses for the 15 FDIs and DNIs knowledge items among clinical nutrition specialists (*N* = 353). The best-recognized interactions were vitamin K–warfarin (78.47% correct), potassium-rich foods with ACE inhibitors (72.52%), and levothyroxine timing relative to meals (64.59%). The least-recognized interactions were grapefruit juice with diltiazem (19.26% correct), albendazole with fatty meals (20.96%), caffeine–theophylline toxicity (21.53%), and phenytoin/phenobarbital-induced folic acid deficiency (28.33%). Among the lower-recognition items, the question on ranitidine and iron absorption (37.68% correct). Ranitidine has been withdrawn from many healthcare markets due to safety concerns, and this item may no longer reflect a clinically current scenario for a portion of respondents.

**Figure 1:**
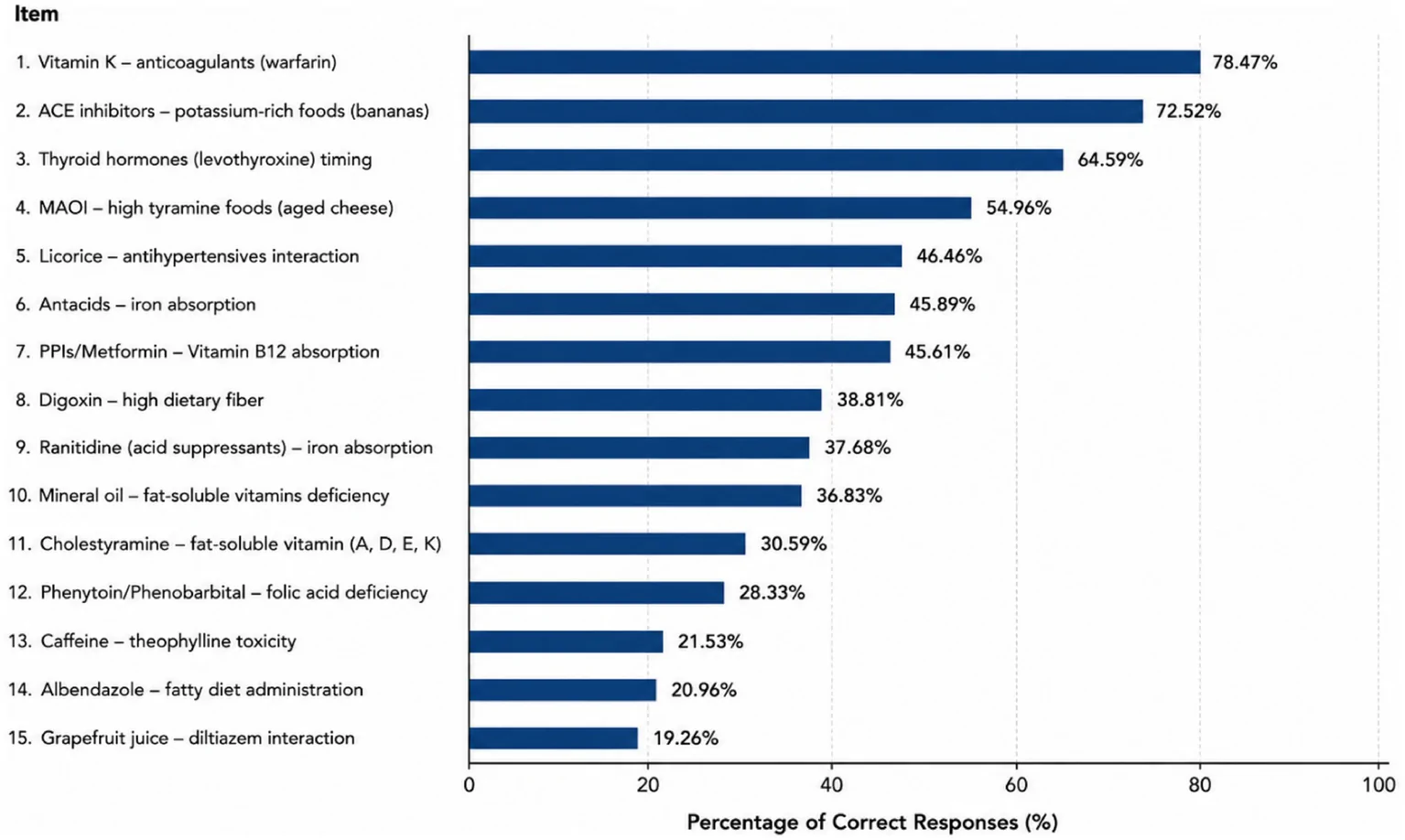
Percentage of correct responses for knowledge items by the study population (*n* = 353).

Figure 2 illustrates the distribution of participants’ attitudes toward FDIs and DNIs among clinical nutrition specialists (*n* = 353). Overall, participants responded selecting “agree” or “strongly agree” across the majority of statements. The strongest positive attitudes were observed for the need to continuously update knowledge about FDIs and DNIs (83.85% agree/strongly agree) and the necessity of knowing about FDIs (83.57%, including 58.36% strongly agreeing). Similarly, high proportions acknowledged the importance of continuously updating knowledge about FDIs and DNIs of medications (83.85%), providing greater attention to these topics during undergraduate studies (77.90%), reporting encountered FDIs and DNIs to pharmacovigilance systems (79.89%), and informing patients about possible FDIs and DNIs as part of professional responsibility (71.10%). Participants also recognized the clinical relevance of FDIs, with 71.01% agreeing that medications can interact with nutrients found in food and 66.01% agreeing that some FDIs can be fatal. In contrast, the statement “I consider that FDIs differ from DNIs” showed the highest level of neutral responses (33.99%) of any item, alongside 32.30% disagreeing or strongly disagreeing, indicating the weakest and most uncertain response pattern in the attitude scale.

**Figure 2:**
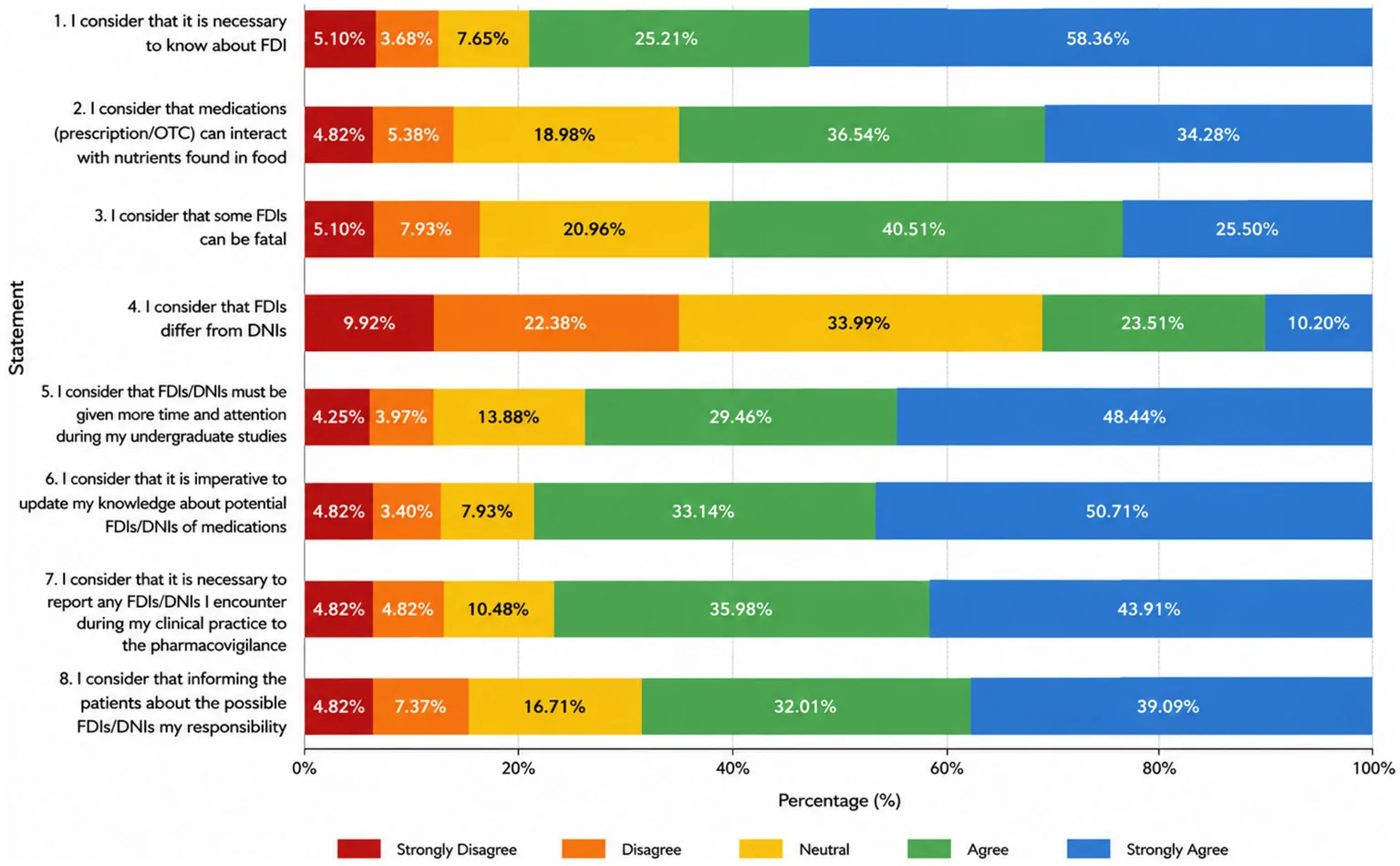
Percentage distribution of responses to FDIs and DNIs statements among the study population (*n* = 353).

Figure 3 illustrates participants’ self-reported practices regarding the management of FDIs and DNIs. The most commonly reported practices were asking patients about their use of medications, supplements, and herbal products (18.98% “usually” and 35.69% “always”) and counseling patients about potential FDIs and DNIs (21.81% “usually” and 35.13% “always”), indicating that more than half of the participants performed these activities routinely. Regular engagement in clinical management activities, including referral, reporting, and documentation of FDIs/DNIs, was reported by 48.16% of participants (“usually” 20.11%, “always” 28.05%). Similarly, 48.16% reported routinely updating their knowledge of FDIs and DNIs (“usually” 20.11%, “always” 28.05%). The use of handbooks or software tools was less consistent, with 42.21% reporting frequent use (“usually” 15.86%, “always” 26.35%) and 31.44% using them sometimes. Consultation of drug information centers was the least frequently reported practice and represented the largest practice gap: only 37.68% reported regular use (“usually” 15.58%, “always” 22.10%).

**Figure 3:**
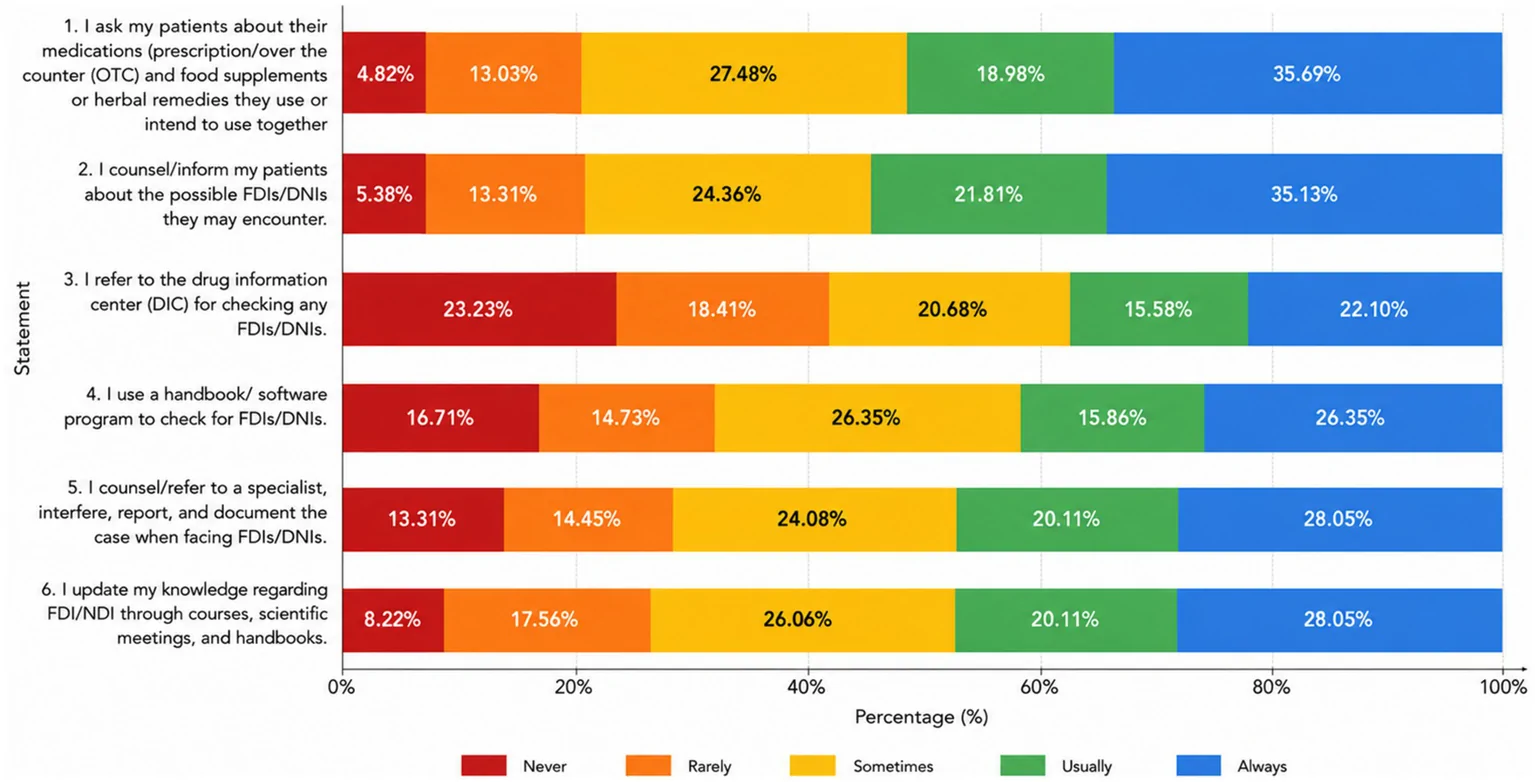
Percentage distribution of responses to practices related to FDIs and DNIs among the study population (*n* = 353).

Table 2 summarizes differences in the mean of KAP scores according to demographic and professional characteristics among clinical nutrition specialists. Overall, most demographic variables were not significantly associated with KAP scores, although several significant differences were identified.

**Table 2:** Analysis of differences in mean knowledge, attitudes, and practices scores across demographic and professional characteristics.

Variables	*n*	Knowledge		Attitudes		Practices	
		Mean ± SD	Test/df/*p*	Mean ± SD	Test/*p*	Mean ± SD	Test/*p*
Gender							
Male	58	6.33 ± 4.20	*t* = 0.194 df = 79.42 *p* = 0.847	30.72 ± 6.68	*t* = −0.743 df = 76.54 *p* = 0.460	20.81 ± 5.91	*t* = −0.741 df = 81.51 *p* = 0.461
Female	295	6.44 ± 4.07		31.43 ± 6.06		20.18 ± 5.97	
Age (years)							
23–29 years	202	6.24 ± 3.86	*F* = 1.885 df = (3, 349) *p* = 0.132	31.48 ± 6.33	*F* = 1.201 df = (3, 349) *p* = 0.309	21.30 ± 5.77	*F* = 5.247 df = (3, 349) *p* = 0.001*
30–39 years	126	6.70 ± 4.39		31.30 ± 5.99		18.67 ± 6.01	
40–49 years	23	6.04 ± 4.12		29.48 ± 5.62		20.22 ± 5.71	
50 years and above	2	12.50 ± 2.12		36.50 ± 0.71		20.00 ± 5.66	
Nationality							
Saudi	325	6.28 ± 4.05	*t* = 2.214 df = 31.48 *p* = 0.034*	31.34 ± 6.17	*t* = −0.308 df = 31.73 *p* = 0.760	20.21 ± 6.00	*t* = 0.818 df = 32.76 *p* = 0.419
Non-Saudi	28	8.11 ± 4.20		30.96 ± 6.23		21.11 ± 5.51	
Geographical area							
Western	133	6.55 ± 4.12	*F* = 0.728 df = (4, 348) *p* = 0.574	32.24 ± 5.57	*F* = 1.903 df = (4, 348) *p* = 0.110	21.08 ± 5.87	*F* = 2.237 df = (4, 348) *p* = 0.065
Eastern	51	6.49 ± 4.00		29.76 ± 7.39		19.41 ± 6.43	
Southern	35	6.31 ± 4.13		30.43 ± 7.28		20.94 ± 6.18	
Central	95	6.67 ± 4.25		30.96 ± 6.49		19.04 ± 5.61	
Northern	39	5.41 ± 3.67		31.82 ± 3.57		21.13 ± 5.85	
Educational qualification							
Bachelor’s	284	6.25 ± 3.90	*F* = 2.878 df = (2, 350) *p* = 0.058	31.33 ± 6.16	*F* = 1.327 df = (2, 350) *p* = 0.267	20.33 ± 5.97	*F* = 0.134 df = (2, 350) *p* = 0.875
Master’s	55	6.69 ± 4.74		31.85 ± 5.78		20.22 ± 6.23	
Doctorate	14	8.86 ± 4.47		28.86 ± 7.44		19.50 ± 4.88	
Graduation year							
Within the last 5 years	212	6.54 ± 4.07	*F* = 0.216 df = (2, 350) *p* = 0.806	31.20 ± 6.54	*F* = 0.089 df = (2, 350) *p* = 0.915	21.37 ± 5.75	*F* = 9.498 df = (2, 350) *p* < 0.001*
5–10 years ago	86	6.30 ± 4.08		31.52 ± 6.09		18.36 ± 5.85	
More than 10 years ago	55	6.18 ± 4.22		31.40 ± 4.68		19.11 ± 6.03	
Graduation university							
Saudi Arabia	310	6.29 ± 4.09	*t* = 1.738 df = 54.95 *p* = 0.088	31.34 ± 6.12	*t* = −0.210 df = 52.63 *p* = 0.834	20.44 ± 5.93	*t* = −1.317 df = 53.64 *p* = 0.193
Non-Saudi	43	7.42 ± 3.99		31.12 ± 6.56		19.14 ± 6.09	
Type of workplace sector							
Government	202	6.20 ± 3.96	*t* = 1.170 df = 310.74 *p* = 0.243	30.64 ± 6.70	*t* = 2.473 df = 350.17 *p* = 0.014*	20.30 ± 6.18	*t* = −0.050 df = 336.67 *p* = 0.960
Private	151	6.72 ± 4.24		32.21 ± 5.25		20.26 ± 5.67	
Years of experience as a clinical nutrition specialist							
0–4 years	245	6.33 ± 4.03	*F* = 1.325 df = (4, 348) *p* = 0.260	31.55 ± 6.05	*F* = 0.477 df = (4, 348) *p* = 0.753	21.17 ± 5.77	*F* = 5.427 df = (4, 348) *p* < 0.001*
5–9 years	60	7.10 ± 4.27		31.17 ± 6.78		18.18 ± 6.29	
10–14 years	35	6.57 ± 3.85		30.29 ± 6.04		17.80 ± 5.61	
15–19 years	9	3.89 ± 4.88		30.67 ± 5.52		21.33 ± 4.85	
Over 20 years	4	6.25 ± 3.77		29.25 ± 7.27		16.75 ± 3.59	
Attended courses on FDI/DNI							
Yes	145	7.37 ± 3.91	*F* = 6.983 df = (2, 350) *p* = 0.001*	30.51 ± 7.29	*F* = 2.897 df = (2, 350) *p* = 0.056	21.13 ± 5.78	*F* = 2.803 df = (2, 350) *p* = 0.062
No	156	5.68 ± 3.99		32.18 ± 5.24		19.51 ± 6.01	
I do not remember	52	6.02 ± 4.40		30.94 ± 4.95		20.23 ± 6.08	
University studies included FDI/DNI subjects							
Yes	251	7.37 ± 3.91	*F* = 10.93 df = (2, 350) *p* < 0.001*	31.17 ± 6.20	*F* = 0.246 df = (2, 350) *p* = 0.782	20.55 ± 5.90	*F* = 1.64 df = (2, 350) *p* = 0.195
No	65	4.68 ± 3.74		31.75 ± 6.45		19.08 ± 6.21	
I do not remember	37	5.27 ± 4.79		31.49 ± 5.49		20.59 ± 5.79	

*t* = independent-samples Welch *t*-test, with degrees of freedom (df) calculated using the Welch–Satterthwaite approximation; *F* = one-way analysis of variance (ANOVA), with degrees of freedom reported as (between-groups df, within-groups df) *p* < 0.05 indicates statistical significance.

For knowledge scores, significant differences were observed according to nationality, attendance of FDIs and DNIs related courses, and inclusion of FDIs and DNIs subjects during university studies. Non-Saudi participants demonstrated significantly higher knowledge scores than Saudi participants (8.11 ± 4.20 vs. 6.28 ± 4.05, *p* = 0.034). Participants who had attended courses on FDIs/DNIs showed significantly higher knowledge scores compared with those who had not attended such courses (7.37 ± 3.91 vs. 5.68 ± 3.99, *p* = 0.001). Similarly, respondents whose university studies included FDI/DNI-related subjects had significantly higher knowledge scores than those without such exposure (7.37 ± 3.91 vs. 4.68 ± 3.74, *p* < 0.001).

Regarding attitudes, clinical nutrition specialists working in the private sector had significantly higher attitude scores compared with those working in the governmental sector (32.21 ± 5.25 vs. 30.64 ± 6.70, *p* = 0.014). No significant differences in attitudes were observed across gender, age, nationality, educational qualification, experience, or educational exposure to FDIs and DNIs.

For practice scores, significant differences were identified according to age, graduation year, and years of professional experience. Participants aged 23–29 years had the highest mean practice score (21.30 ± 5.77), compared with 18.67 ± 6.01 among those aged 30–39 years (*p* = 0.001). Practice scores also varied significantly by graduation year (*p* < 0.001), with the highest score among those who graduated within the last 5 years (21.37 ± 5.75). Similarly, practice scores differed significantly according to years of professional experience (*p* < 0.001), with the highest score among those with 0–4 years of experience (21.17 ± 5.77). No significant differences in practice scores were observed across gender, nationality, geographical area, educational qualification, graduation university, workplace sector, or university studies.

### 3.3 Factors associated with nutritional knowledge, attitude, and practice scores

Figures 4–6 present the adjusted associations between participant characteristics and knowledge, attitude and practice scores, respectively. In Figure 4, the forest plot displays significant independent factors of higher nutritional knowledge among clinical nutrition specialists. Holding a Doctorate (*β* = 2.43, 95% CI: 0.36–4.51) is associated with a significant increase in knowledge scores compared to a bachelor’s degree. Formal training (*β* = 1.15, 95% CI: 0.24–2.05) and university coursework (*β* = 2.14, 95% CI: 1.06–3.21) on food-drug interactions are both strongly associated with higher knowledge. Additionally, clinical nutrition specialists who manage patients with Digestive Diseases or cardiovascular diseases show modestly higher knowledge scores (*β* ≈ 1.03–1.04). The model explains 11.5% of the variance (Adjusted *R*^2^ = 0.115).

**Figure 4:**
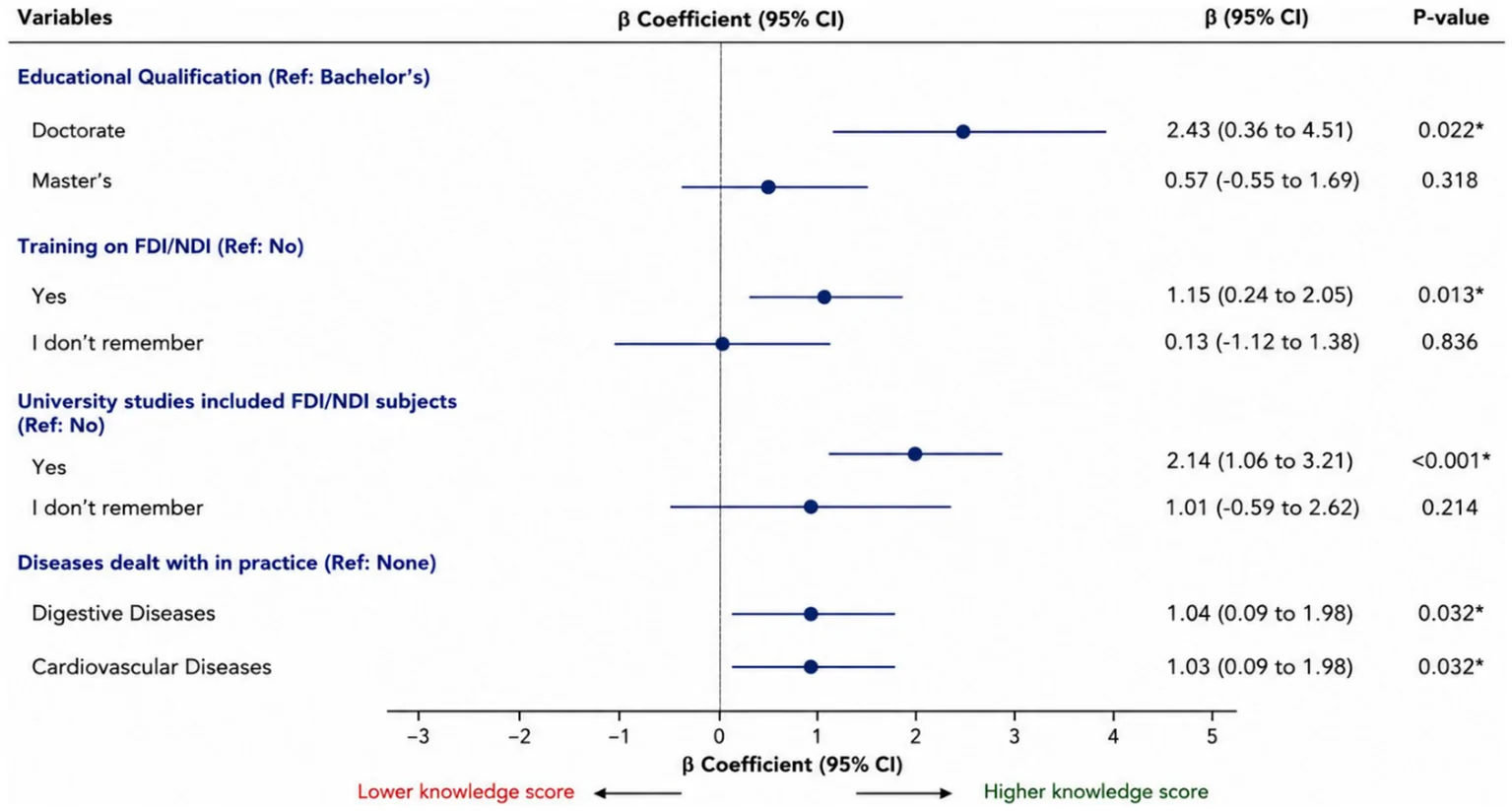
Multivariable linear regression analysis of factors associated with knowledge scores regarding FDIs and DNIs among the study population (*N* = 353). Points represent adjusted *β* coefficients and horizontal lines represent 95% confidence intervals. Ref, Reference category; CI, Confidence interval; Adjusted *R*^2^ = 0.115; *p < 0.05.

**Figure 5:**
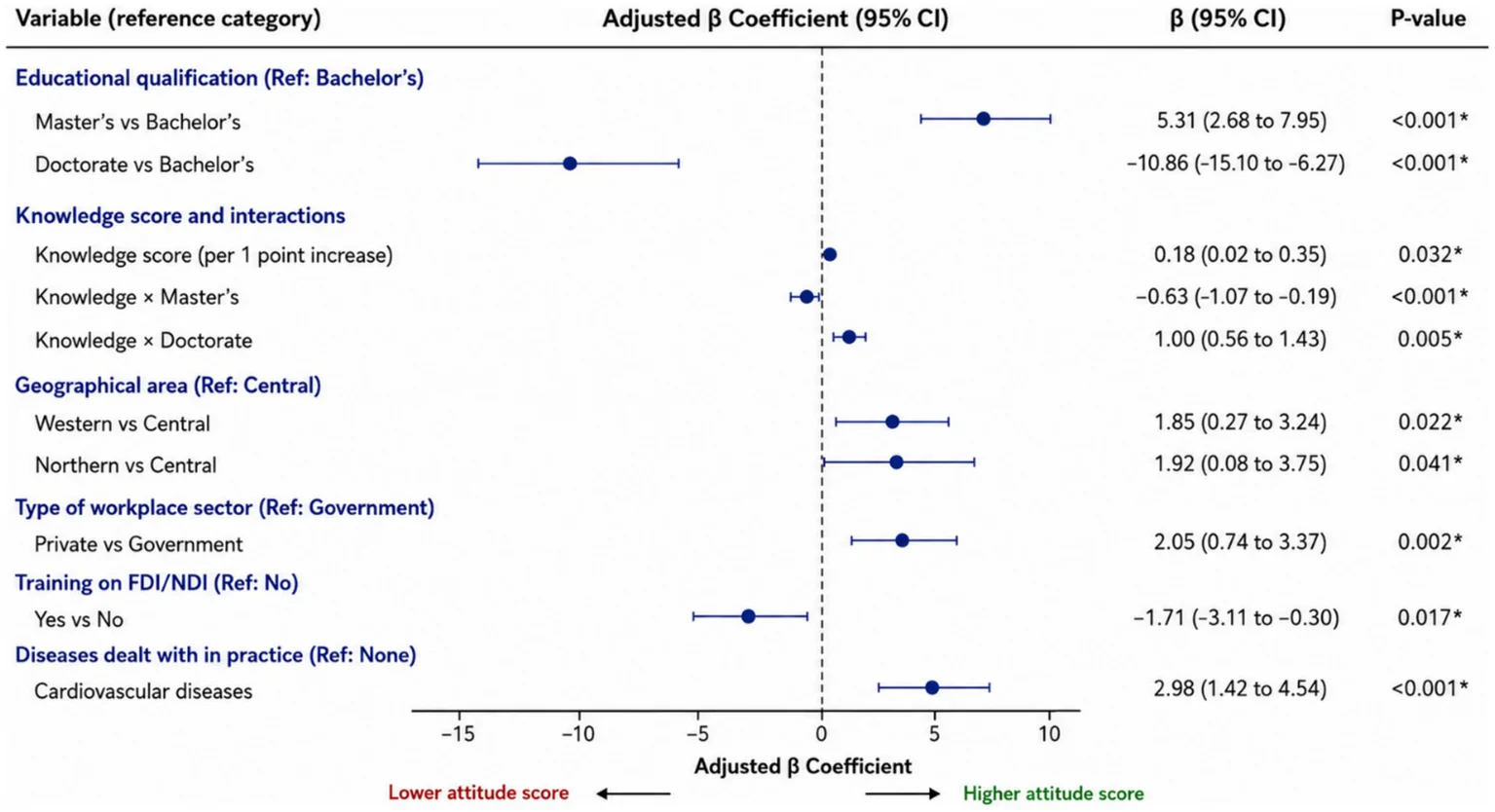
Multivariable linear regression analysis of factors associated with attitude scores regarding FDIs and DNIs among the study population (*N* = 353). Points represent adjusted *β* coefficients and horizontal lines represent 95% confidence intervals. Ref, Reference category; CI, Confidence interval; Adjusted *R*^2^ = 0.134; *p < 0.05.

**Figure 6:**
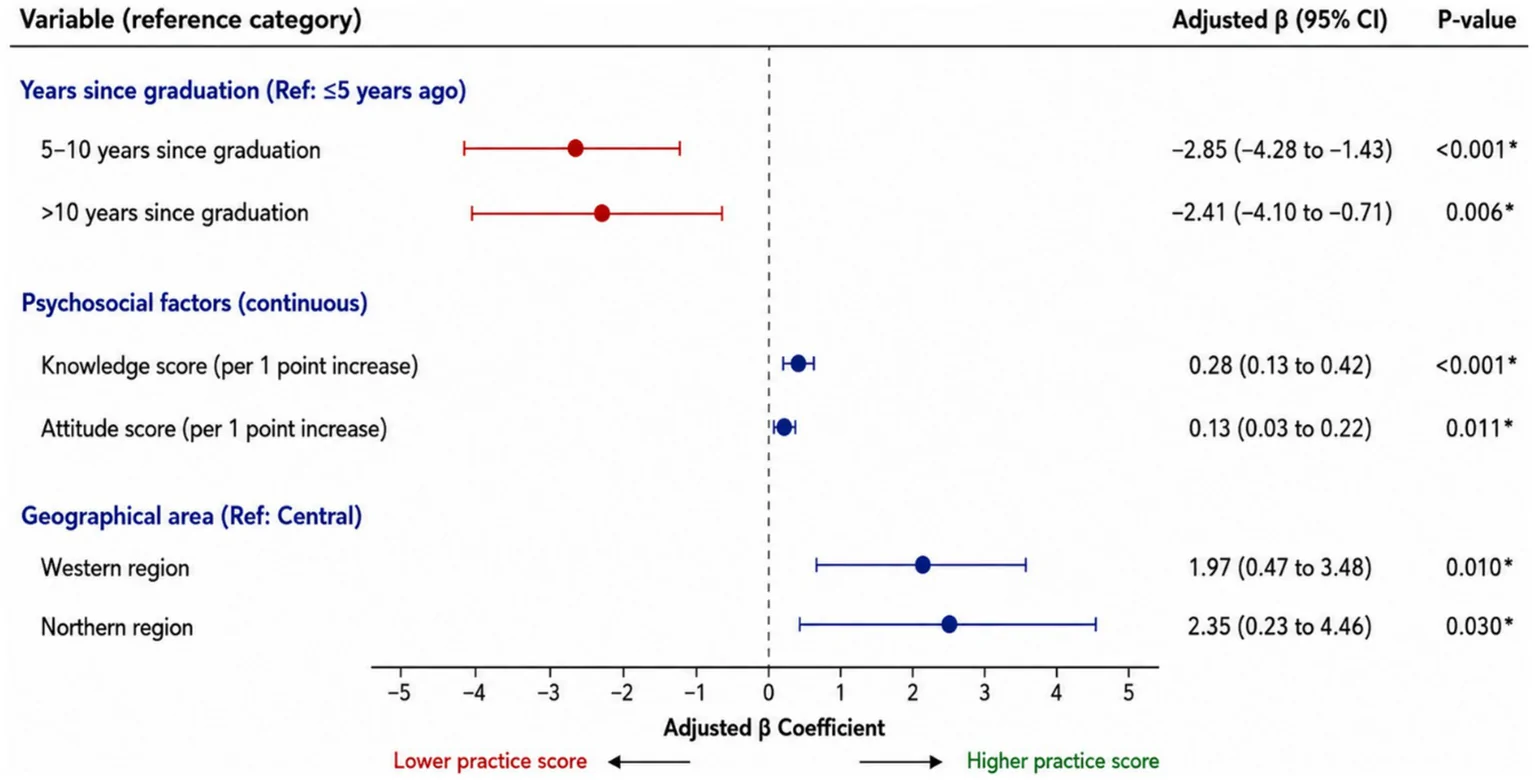
Multivariable linear regression analysis of factors associated with practice scores regarding FDIs and DNIs among the study population (*N* = 353). Points represent adjusted *β* coefficients and horizontal lines represent 95% confidence intervals. Ref, Reference category; CI, Confidence interval; Adjusted *R*^2^ = 0.128; *p < 0.05.

Figure 5 demonstrates that educational qualification was significantly associated with attitudes, although the relationship varied according to knowledge level. Compared with participants holding a bachelor’s degree, those with a master’s degree had significantly higher attitude scores (*β* = 5.31, 95% CI: 2.68–7.95; *p* < 0.001), whereas those with a doctoral degree had significantly lower attitude scores (*β* = −10.86, 95% CI: −15.10 to −6.27; *p* < 0.001). Participants practicing in the Western (*β* = 1.85, 95% CI: 0.27–3.24; *p* = 0.022) or Northern (*β* = 1.92, 95% CI: 0.08–3.75; *p* = 0.041) regions, those employed in the private sector (*β* = 2.05, 95% CI: 0.74–3.37; *p* = 0.002), and those managing patients with cardiovascular diseases (*β* = 2.98, 95% CI: 1.42–4.54; *p* < 0.001) had significantly higher attitude scores than their respective reference groups. In contrast, previous attendance at FDIs and DNIs training courses was associated with lower attitude scores (*β* = −1.71, 95% CI: −3.11 to −0.30; *p* = 0.017). Overall, the final model explained 13.4% of the variability in attitude scores (adjusted *R*^2^ = 0.134).

The plot in Figure 6 highlights that a longer time since graduation was associated with lower practice scores. Compared to recent graduates (≤5 years), those who graduated 5–10 years ago (*β* = −2.85) and over 10 years ago (*β* = −2.41) had significantly lower practice scores. Conversely, higher knowledge scores (*β* = 0.28) and higher attitude scores (*β* = 0.13) were each independently associated with higher practice scores. Geographically, clinical nutrition specialists in the Western (*β* = 1.97) and Northern (*β* = 2.35) regions report better practices than those in the Central region. The model explains 12.8% of the variance (Adjusted *R*^2^ = 0.128). Regression diagnostic analyses did not identify any important violations of linearity, homoscedasticity, normality of residuals, or influential observations that would affect the validity of the final models.

## 4 Discussion

### 4.1 Sociodemographic influences on KAP

This study provides the first comprehensive assessment of clinical nutrition specialists’ KAP regarding FDIs and DNIs in Saudi Arabia. The predominance of female participants is consistent with the gender distribution previously described for the dietetic workforce in Saudi Arabia and the wider Gulf region (28). This likely reflects the composition of the profession rather than sampling bias and suggests that the study population is broadly representative of the current clinical nutrition workforce.

Knowledge scores were relatively consistent across most sociodemographic variables, suggesting that the baseline understanding of FDIs and DNIs was broadly comparable among clinical nutrition specialists in this sample. This finding is in line with prior KAP studies indicating that when educational exposure is similar, demographic variables are only weakly associated with core knowledge (29). However, nationality and prior exposure to FDIs and DNIs-related education were significantly associated with knowledge levels. Participants who reported formal training achieved higher scores, consistent with evidence that structured education contributes to greater competence in this domain (30). Variability in nutrition curricula across institutions in Saudi Arabia could plausibly explain part of these differences, although this was not directly assessed in the present study. Attitude scores were relatively high across all subgroups. The slightly higher attitude scores observed among private-sector clinical nutrition specialists were not accompanied by data on the professional environment or access to continuing education; therefore, this difference should be interpreted cautiously and regarded as an observation requiring further investigation rather than a finding with a clear explanation. Practice behaviors were significantly associated with age, years since graduation, and professional experience, with younger and recently trained clinical nutrition specialists reporting better practices. This pattern has been described in other healthcare studies where recent graduates demonstrate closer alignment with current evidence-based guidelines and greater use of digital tools (31).

### 4.2 Knowledge, attitudes, and practices regarding FDIs and DNIs

Overall, the findings revealed that the mean knowledge (42.9% of the maximum possible score) and practice (67.5% of the maximum possible score) scores were well below their respective maxima, whereas the mean attitude score was closer to its maximum (78.5%). These results suggest the presence of a knowledge–practice gap among the study sample. Similar discrepancies between awareness and clinical implementation have been reported in previous studies, where contextual and organizational barriers are may hinder the translation of knowledge into practice (32, 33). Participants demonstrated better recognition of common, clinically emphasized interactions such as the vitamin K–warfarin, potassium-rich foods with ACE inhibitors, and levothyroxine administration timing. This is consistent with findings suggesting that frequently encountered drug–food interactions are more often recognized, likely because they are routinely incorporated into undergraduate training, clinical guidelines, and everyday practice (2, 33, 34). In contrast, knowledge of more specialized interactions, including grapefruit juice and calcium channel blockers, caffeine–theophylline toxicity, and the effects of food on drug absorption, was substantially lower. This finding is consistent with reports of persistent deficiencies in understanding pharmacokinetic drug–food interactions, particularly those involving cytochrome P450-mediated metabolism, which are generally more complex and may receive less emphasis during professional education and continuing training (33, 35). A recent Turkish study similarly found that healthcare professionals demonstrated moderate knowledge of food–drug interactions (median score: 13/21), with professional discipline—particularly pharmacists, who achieved the highest scores—emerging as the strongest predictor of knowledge (36), Together, these findings suggest that differences in educational preparation and professional training, rather than demographic characteristics alone, play a key role in shaping competency in managing food–drug interactions across healthcare settings. Furthermore, the finding that a substantial proportion of participants were unable to clearly distinguish between FDIs and DNIs indicates an important conceptual gap. Addressing this distinction through undergraduate curricula and continuing professional development may improve both clinical understanding and the appropriate identification and management of these interactions in practice.

Attitudes were relatively high (78.5% of the maximum possible score), reflecting clinical nutrition specialists’ recognition of the importance of FDIs and DNIs. According to the Theory of Planned Behavior (TPB), attitudes represent one of the key determinants of behavioral intention (37, 38). However, some conceptual ambiguity persisted regarding the distinction between FDIs and DNIs, suggesting a discrepancy between general awareness and conceptual understanding. Similar gaps have been reported in previous studies, highlighting that healthcare professionals may recognize the clinical importance of these interactions without fully understanding their underlying concepts or classifications (33, 39).

Practice levels averaged 67.5% of the maximum possible score, with inconsistencies in the implementation of recommended practices. Although many clinical nutrition specialists reported routinely asking patients about medication use and providing counseling, fewer consistently used drug information resources or clinical decision-support tools. The underuse of these resources has been attributed in other healthcare settings to organizational barriers, heavy workloads, limited access to resources, and inadequate integration of decision-support systems into routine clinical workflows (33, 40). However, whether these factors contributed to the observed practices in the present study cannot be determined because they were not directly assessed. Recently graduated clinical nutrition specialists reported significantly better practice scores than their more experienced counterparts, which is consistent with evidence suggesting that early-career healthcare professionals are more likely to adopt evidence-based practices and digital clinical tools (41). Collectively, these findings suggest the presence of a knowledge–practice gap, whereby positive attitudes and moderate knowledge do not consistently translate into optimal clinical practice. Nevertheless, because of the cross-sectional study design, these findings should be interpreted as associations rather than evidence of a causal relationship between knowledge, attitudes, and practice.

### 4.3 Factors associated with KAP: multivariable analysis insights

The present study identified several factors significantly associated with knowledge, attitude, and practice regarding FDIs and DNIs, although the direction and consistency of these associations differed by domain. Educational level and formal training were consistently associated with greater knowledge but showed an inconsistent, and for prior training and doctoral-level education, inverse relationship with attitude, and neither was independently associated with practice. Clinical exposure to digestive or cardiovascular disease patients was associated with both higher knowledge and more positive attitudes, while workplace sector and regional differences were associated with attitude and practice scores, respectively. Because the design was cross-sectional, these relationships should be interpreted as associations. Similarly, the positive associations of knowledge and attitude scores with practice scores identified in the multivariable practice model are presented as consistent with, but not confirming, the sequential KAP framework, in which knowledge, attitude, and practice are assumed to build on one another. Clinical nutrition specialists holding doctoral degrees had significantly higher knowledge scores than those with bachelor’s degrees, which is consistent with previous work linking advanced academic training to stronger evidence-based practice skills and greater exposure to specialized clinical nutrition content, including pharmaconutrition (3, 42). The association between university coursework and higher knowledge scores similarly supports the value of integrating FDIs and DNIs into undergraduate curricula, which is consistent with findings among clinical nutrition specialists and nursing professionals (34, 43).

Formal training on FDIs/DNIs was also associated with higher knowledge scores, consistent with the broader literature on continuing professional education (44). Clinical nutrition specialists who routinely managed patients with digestive or cardiovascular diseases also had higher knowledge levels, which may reflect greater clinical exposure to relevant therapeutic regimens, although this study did not directly measure the nature or intensity of that exposure.

Findings for attitudes were more complex. Master’s degree holders reported significantly more positive attitudes than bachelor’s degree holders, whereas doctorate holders reported lower attitude scores despite higher knowledge. Because the doctorate subgroup comprised relatively few participants, this finding should be interpreted with caution. Small subgroup sizes can produce unstable regression coefficients, wider confidence intervals, and less precise effect estimates, making subgroup comparisons more susceptible to sampling variability (45–47). Consequently, this finding should be regarded as exploratory rather than definitive and requires confirmation in larger studies including a greater representation of doctoral-level practitioners. A tentative interpretation is that more specialized professionals may adopt a more critical stance toward FDIs/DNIs due to their greater awareness of the complexity, uncertainty, and clinical challenges involved in managing these interactions. However, this explanation is speculative and was not directly tested; similar knowledge–attitude discrepancies have been reported elsewhere in healthcare education (48).

Participation in training courses was associated with lower, rather than higher, attitude scores. This inverse association should also be interpreted cautiously given its counterintuitive direction. Several explanations are possible, including residual confounding, suppression effects arising from correlated predictors, response bias, or model instability. One possible, but untested, explanation is that trained clinical nutrition specialists may become more aware of the complexity and clinical risks associated with FDIs/DNIs, which could lead to more cautious rather than more confident attitudes. Comparable patterns, whereby greater knowledge is accompanied by more critical self-assessment rather than increased confidence, have been suggested in previous healthcare education studies (34), although this mechanism was not directly evaluated in the present study. Therefore, this finding should be interpreted as hypothesis-generating rather than evidence of a causal relationship.

Clinical nutrition specialists working in private healthcare settings and in the Western and Northern regions reported more positive attitudes and better practices than those in the Central region. These regional and sector differences were not accompanied by direct data on institutional resources, continuing-education access, or workload in this study, and should therefore be reported descriptively as findings requiring further investigation rather than explained by these unmeasured factors. Years since graduation were negatively associated with practice scores, with older graduates reporting lower practice performance than recent graduates, which may reflect greater familiarity among recent graduates with contemporary evidence-based guidelines, digital health technologies, and clinical decision-support tools incorporated into modern nutrition education (41). However, because digital literacy, guideline use, and continuing professional development were not directly assessed, this explanation remains speculative. Higher knowledge and attitude scores were independently associated with better practice scores, providing empirical support for the theoretical assumptions of the KAP framework. However, these associations should not be interpreted as evidence of a causal pathway because of the cross-sectional study design. The adjusted *R*^2^ values of the three models remained modest (11.5–13.4%), indicating that other, unmeasured factors likely contribute to KAP regarding FDIs and DNIs, such as workload, interdisciplinary communication, organizational support, guideline accessibility, self-efficacy, and institutional culture. Future studies, particularly longitudinal and mixed-methods investigations, are needed to explore these contextual determinants.

In summary, the present findings underscore the value of strengthening academic curricula, promoting continuing professional education, and developing targeted interventions to support clinical nutrition specialists’ competencies regarding FDIs and DNIs, including improving access to evidence-based drug information resources and clinical decision-support tools, thereby enhancing patient safety and the quality of nutrition care.

## 5 Limitations

This study has several limitations. First, the cross-sectional design precludes any inference of causality between knowledge, attitudes, and practices; associations reported here should not be interpreted as evidence of a causal or temporal sequence (49, 50).

Second, the study used online convenience sampling, which may limit the representativeness of the findings. Recruitment did not rely on a defined sampling frame as there was no fixed denominator (total number of clinical nutrition specialists invited or eligible). Furthermore, the number of eligible clinical nutrition specialists who received or viewed the survey invitation was unknown. Hence, the response rate could not be calculated, limiting the assessment of potential non-response bias. Third, the sample was characterized by an overrepresentation of young, female, bachelor’s-level, and early-career clinical nutrition specialists. This demographic skew may have inflated estimates of positive practice behaviors (since younger/recent graduates reported better practices) and limits the generalizability of the findings to more senior, male, or postgraduate-level clinical nutrition specialists, or to those recruited through non-online channels. This type of sampling is known to affect representativeness by potentially excluding less engaged or less accessible professionals (51). Fourth, the reliance on self-reported data for both attitudes and practices may introduce social desirability bias, particularly for practice-related items: participants may overreport desirable behaviors, such as counseling patients or updating their knowledge, while underreporting less favorable practices. Self-reported measures of professional behavior have been shown to overestimate actual practice relative to direct observation (52, 53), which may partly explain the gap observed here between positive attitudes and moderate practice levels. Finally, although the questionnaire was adapted from a previously validated instrument and underwent expert face and content validation with satisfactory internal consistency, formal psychometric validation (e.g., exploratory or confirmatory factor analysis, convergent and discriminant validity, or test–retest reliability) was not conducted following the modifications. Consequently, the construct validity of the adapted instrument cannot be fully established, as content validity and internal consistency alone are insufficient to demonstrate construct validity after instrument modification (22). Future studies should undertake a comprehensive psychometric evaluation of the adapted questionnaire before its broader application.

## 6 Conclusion

This study provides the first comprehensive assessment of clinical nutrition specialists’ KAP regarding FDIs and DNIs in Saudi Arabia. Although mean attitude scores were relatively high (78.5% of the maximum possible score), mean knowledge (42.9%) and practice (67.5%) scores indicated important gaps relative to the maximum possible scores. Higher educational attainment, prior FDI/DNI training, and university education on these topics were each associated with greater knowledge; however, their relationship with attitudes differed by domain and by qualification level, training and doctoral-level education were associated with lower attitude scores, while a Master’s degree was associated with higher scores, and none of these factors remained independently associated with practice once knowledge and attitude were accounted for. These findings highlight the importance of strengthening undergraduate curricula and examining why advanced training may not translate into more favorable attitudes, while expanding continuing professional development. Improving access to drug information resources and integrating clinical decision-support tools into routine practice may further enhance the identification and management of FDIs and DNIs, ultimately contributing to safer and more effective patient care.

## Data Availability

The raw data supporting the conclusions of this article will be made available by the authors, without undue reservation.
