# Supplementary material for: Factors associated with knowledge, attitudes, and practices regarding food-drug interactions and drug-nutrient interactions among clinical nutrition specialists in Saudi Arabia
Source: Front Public Health. 2026 Sep 21;14:1914823. doi: 10.3389/fpubh.2026.1914823 (PMC13635382; doi:10.3389/fpubh.2026.1914823)
Supplement: Supplementary file 1 [file Data_Sheet_1.pdf]

Food drug / drug-nutrient interactions questionnaire for Dietitians in the Kingdom of Saudi Arabia:
(FDIQ, DNIQ)

A. Socio-demographics:

1. Gender:
   - ☐ Male
   - ☐ Female
2. Age:
   - ☐ 23-29 years
   - ☐ 30-39 years
   - ☐ 40-49 years
   - ☐ 50 years and above
3. Nationality:
   - ☐ Saudi
   - ☐ Non-Saudi (please specify): _
4. Geographical area:
   - ☐ Western region
   - ☐ Eastern region
   - ☐ Southern region
   - ☐ Central region
   - ☐ Northern region
5. Educational Qualification:
   - ☐ Bachelor's
   - ☐ Master's
   - ☐ Doctorate
6. Graduation Year:
   - ☐ Before 5 years ago.
   - ☐ 5-10 years ago.
   - ☐ More than 10 years ago.
7. Graduation university:
   - ☐ Saudi Arabia
   - ☐ non-Saudi

10. Type of Workplace sector:
    - ☐ Government
    - ☐ Private
11. Years of Experience as a dietitian:
    - ☐ 0-4
    - ☐ 5-9

- ☐ 10-14
- ☐ 15-19
- ☐ Over 20

12. Have you attended courses on food-drug interactions and Nutrient-drug interaction?

- ☐ Yes
- ☐ No
- ☐ I don't remember

13. Did your university studies include subjects related to food-drug interactions and Nutrient-drug interaction?

- ☐ Yes
- ☐ No
- ☐ I don't remember

14. What diseases do you deal/delt with in your practice as a dietitian:

- ☐ Digestive Diseases such as Peptic Ulcer,Ulcerative Colitis, IBS, Celiac disease
- ☐ Endocrine Diseases such as (Thyroid , Diabetes)
- ☐ cardiovascular Disease as (Hypertension,.....)
- ☐ Inherited Metabolic Disorders
- ☐ Food Allergies
- ☐ Mental Health
- ☐ kidney diseases
- ☐ Cancer
- ☐ Other…….

B. Knowledge about the most common FDIs and DNIs:

1) Which nutrient found in green leafy vegetables, liver, and green tea can significantly interfere with anticoagulants as warfarin?
   1-Vitamin C 2-**Vitamin K** 3-VitaminB12 4- Don't know
2) Caffeine found in coffee, tea, and chocolate can increases the risk of toxicity from the following drug(s):
   1- Diazepam 2- Pseudoephedrine 3- **Theophylline** 4- Don't know
3) The following is preferred to be taken with fatty diet to maximize its effect:
   1- cycloserine **2- Albendazole** 3- Penicillins 4- Don't know
4) Licorice is known to interfere with the following therapy:
   1- **Antihypertensives** 2- Anti-inflammatory 3- Antihistamines 4- Don't know
5) The following is known to interfere with Digoxin therapy :
   1- **High dietary fibers** 2- carbohydrate 3- Proteins 4-Don't know
6) Patients on antidepressant therapy as MAO Inhibitors should avoid the following food as it contains high amounts of tyramine amino acid:
   1-Aged cheese **2-** Bread 3-Rice 4- Don't know

7) Patients on ACE inhibitors as captopril should be advised to avoid food rich in potassium as :
1- Apples **2-Bananas** **3- blueberry** 4- Don't know

8) Grapefruit juice interferes with the metabolism of the following drug(s):
1- Acetaminophen **2-Diltiazem** **3-** Pravastatin 4- Don't know

9) Regarding food intake, the best way to benefit from thyroid hormones as levothyroxin is to be taken:
1- **Before meals** 2- After meals 3-With meals 4- Don't know

10) PPIs as omeprazole and antidiabetics as metformin disturb the active absorption of which of the following?
1-Vitamin B12 2-Vitamin A 3-vitamin B6 4- Don't know

11) Which of the following drug(s) results in a decrease in the absorption of fat soluble vitamins (A, D, E&K)?
1-Hydrochlorothiazide 2-Prednisone **3-Cholestyramine** 4- Don't know

12) The chronic administration of phenytoin and phenobarbital can cause deficiency of the following:
1-**Folic acid &** 2-Riboflavin 3- pyridoxine 4- Don't know

13) Acid-suppressing medications as ranitidine can decrease the absorption of:
1-Folic acid **2-Iron** 3-Vitamin D3 4-Don't know

14) The chronic use of mineral oil as a laxative is often associated with deficiencies of:
1-Water soluble vitamins **2-Fat soluble vitamins** 3- Proteins 4- Don't know

15) The following medication(s) can interfere with iron absorption:
1-Antacids 2-Flagyl 3-Augmentin 4- Don't know

C. Attitude towards FDIs and DNIs:

1. I consider that it is necessary to know about FDI:
1. Strongly Disagree 2. Disagree 3. Neutral 4. Agree 5. Strongly Agree

2. I consider that medications (prescription/OTC) can interact with nutrients found in food:
1. Strongly Disagree 2. Disagree 3. Neutral 4. Agree 5. Strongly Agree

3. I consider that some FDIs can be fatal:
1. Strongly Disagree 2. Disagree 3. Neutral 4. Agree 5. Strongly Agree

4. I consider that FDIs differ from DNIs:
1. Strongly Disagree 2. Disagree 3. Neutral 4. Agree 5. Strongly Agree

5. I consider that FDIs/DNIs must be given more time and attention during my undergraduate studies:
1. Strongly Disagree 2. Disagree 3. Neutral 4. Agree 5. Strongly Agree

6. I consider that it is imperative to update my knowledge about potential FDIs/DNIs of medications:
1. Strongly Disagree 2. Disagree 3. Neutral 4. Agree 5. Strongly Agree

7. I consider that it is necessary to report any FDIs/DNIs I encounter during my clinical practice to the pharmacovigilance:
1. Strongly Disagree 2. Disagree 3. Neutral 4. Agree 5. Strongly Agree

8. I consider that informing the patients about the possible FDIs/DNIs my responsibility:
1. Strongly Disagree 2. Disagree 3. Neutral 4. Agree 5. Strongly Agree

<u>Practice towards FDIs and DNIs:</u>

Question	Never	Rarely	Sometimes	Usually	Always
1. I ask my patients about their medications (prescription/over the counter (OTC) and food supplements or herbal remedies they use or intend to use together:					
2. I counsel/inform my patients about the possible FDIs/DNIs they may encounter:					
3. I refer to the drug information center (DIC) for checking any FDIs/DNIs:					
4. I use a handbook/ software program to check for FDIs/DNIs:					
5. I counsel/refer to a specialist, interfere, report, and document the case when facing FDIs/DNIs.					
6. I update my knowledge regarding FDI/NDI through courses, scientific meetings, and handbooks.					
