# Supplementary material for: Factors associated with knowledge, attitudes, and practices regarding food-drug interactions and drug-nutrient interactions among clinical nutrition specialists in Saudi Arabia
Source: Front Public Health. 2026 Sep 21;14:1914823. doi: 10.3389/fpubh.2026.1914823 (PMC13635382; doi:10.3389/fpubh.2026.1914823)
Supplement: Supplementary file 2 [file Data_Sheet_2.pdf]

Supplementary Regression Tables

Independent re-analysis of the supplied raw dataset (N=353), using the final model specifications reported in the original biostatistical results. Standard errors and 95% CIs are reported for all final-model coefficients. HC3 heteroscedasticity-robust standard errors were used for the attitude model because the residual diagnostics indicated heteroscedasticity; this reproduces the CIs reported in the original results.

Supplementary Table S1. Knowledge-score regression

Initial model: nationality; geographical area; educational qualification; graduation university; years of experience; FDI/DNI training; university FDI/DNI coursework; endocrine disease; cardiovascular disease; inherited metabolic disorders; kidney disease; obesity; cancer. Variables removed during backward elimination: nationality, geographical area, graduation university, years of experience, endocrine disease, inherited metabolic disorders, kidney disease, obesity, and cancer.

Variable	Reference	β	SE	95% CI	p-value	VIF
Master's degree	Bachelor's	0.57	0.57	-0.55 to 1.69	0.3178	1.02
Doctorate	Bachelor's	2.43	1.06	0.36 to 4.51	0.0217	1.01
FDI/DNI training: Yes	No	1.15	0.46	0.24 to 2.05	0.0131	1.22
FDI/DNI training: I don't remember	No	0.13	0.64	-1.12 to 1.38	0.8359	1.21
University FDI/DNI coursework: Yes	No	2.14	0.55	1.06 to 3.21	0.0001	1.47
University FDI/DNI coursework: I don't remember	No	1.01	0.82	-0.59 to 2.62	0.2143	1.49
Digestive disease	No	1.04	0.48	0.09 to 1.98	0.0317	1.24
Cardiovascular disease	No	1.03	0.48	0.09 to 1.98	0.0323	1.21

Analytic n=353; adjusted R^2=0.115; maximum individual VIF=1.492. Diagnostics: Shapiro–Wilk p=5.12e-07; Breusch–Pagan p=0.395; maximum Cook's distance=0.044; maximum leverage=0.118; standardized residuals |r|>3: 0 observations.

Supplementary Table S2. Attitude-score regression

Initial main-effects model (p<0.20 screening): geographical area; educational qualification; knowledge score; workplace sector; FDI/DNI training; digestive disease; endocrine disease; cardiovascular disease; food allergies; kidney disease; obesity. The education×knowledge interaction terms were evaluated in the attitude model and retained in the final model. Variables removed during backward elimination: digestive disease, endocrine disease, food allergies, kidney disease, and obesity. HC3 robust SEs were used for the final attitude model.

Variable	Reference	β	SE	95% CI	p-value	VIF
Master's degree	Bachelor's	5.31	1.34	2.68 to 7.95	<0.0001	3.43
Doctorate	Bachelor's	-10.86	2.24	-15.10 to -6.27	<0.0001	5.41
Western region	Central region	1.85	0.80	0.27 to 3.43	0.0222	1.54

Eastern region	Central region	-0.71	1.20	-3.07 to 1.66	0.5569	1.36
Southern region	Central region	0.52	1.39	-2.21 to 3.24	0.7106	1.28
Northern region	Central region	1.92	0.93	0.08 to 3.75	0.0409	1.31
FDI/DNI training: Yes	No	-1.71	0.72	-3.12 to -0.30	0.0177	1.22
FDI/DNI training: I don't remember	No	-1.20	0.80	-2.79 to 0.38	0.1354	1.17
Knowledge score	—	0.18	0.09	0.02 to 0.35	0.0322	1.44
Master's × knowledge	Bachelor's × knowledge	-0.63	0.23	-1.07 to -0.19	0.0054	3.64
Doctorate × knowledge	Bachelor's × knowledge	1.00	0.22	0.57 to 1.43	<0.0001	5.59
Private sector	Government	2.05	0.67	0.74 to 3.37	0.0023	1.08
Cardiovascular disease	No	2.98	0.79	1.42 to 4.54	0.0002	1.07

Analytic n=353; adjusted R^2=0.134; maximum individual VIF=5.593. Diagnostics: Shapiro–Wilk p=4.75e-13; Breusch–Pagan p=0.00015; maximum Cook's distance=0.091; maximum leverage=0.283; standardized residuals |r|>3: 8 observations.

Supplementary Table S3. Practice-score regression

Initial model (p<0.20 screening): age; geographical area; graduation year; graduation university; years of experience; FDI/DNI training; university FDI/DNI coursework; inherited metabolic disorders; obesity; knowledge score; attitude score. Variables removed during backward elimination: age, graduation university, years of experience, FDI/DNI training, university FDI/DNI coursework, inherited metabolic disorders, and obesity.

Variable	Reference	β	SE	95% CI	p-value	VIF
5–10 years since graduation	≤5 years	-2.85	0.72	-4.28 to -1.43	<0.0001	1.08
>10 years since graduation	≤5 years	-2.41	0.86	-4.10 to -0.71	0.0055	1.09
Western region	Central region	1.97	0.76	0.47 to 3.48	0.0103	1.53
Eastern region	Central region	0.77	0.98	-1.16 to 2.69	0.4349	1.33
Southern region	Central region	1.54	1.12	-0.66 to 3.74	0.1686	1.25
Northern region	Central region	2.35	1.08	0.23 to 4.46	0.0299	1.27
Knowledge score	—	0.28	0.07	0.13 to 0.42	0.0002	1.02
Attitude score	—	0.13	0.05	0.03 to 0.22	0.0109	1.03

Analytic n=353; adjusted R^2=0.128; maximum individual VIF=1.534. Diagnostics: Shapiro–Wilk p=0.021; Breusch–Pagan p=0.938; maximum Cook's distance=0.030; maximum leverage=0.071; standardized residuals |r|>3: 0 observations.
